# Development of a New Micropropagation Protocol and Transfer of In Vitro Plants to In Vivo Conditions for Cascade Hop

**DOI:** 10.3390/plants12152877

**Published:** 2023-08-06

**Authors:** Nicolò Iacuzzi, Francesco Salamone, Davide Farruggia, Noemi Tortorici, Lorena Vultaggio, Teresa Tuttolomondo

**Affiliations:** Department of Agricultural, Food and Forest Sciences, Università Degli Studi di Palermo, Viale delle Scienze 13, Building 4, 90128 Palermo, Italy; nicolo.iacuzzi@unipa.it (N.I.); francesco.salamone08@unipa.it (F.S.); noemi.tortorici@you.unipa.it (N.T.); lorena.vultaggio@unipa.it (L.V.); teresa.tuttolomondo@unipa.it (T.T.)

**Keywords:** *Humulus lupulus*, micropropagation, single-node explants, cytokinin, auxins

## Abstract

The vegetative propagation of hops, despite being a reliable method, is not very common due to the unavailability of the plant material. In this study, the technique of in vitro propagation was applied to the Cascade variety of *Humulus lupulus* L. The plant material was collected from a private field in Sicily; the explants were subjected to sterilization before in vitro culture. Single-node explants were placed in in vitro culture in nine different culture media for multiplication. Thidiazuron (TDZ), Benzyladenine (BAP) and meta-Topoline (mT) were tested for multiplication phase. For the rooting phase, five types of different culture media were evaluated. Binodal cuttings coming from the previous multiplication test were placed in the culture. The rooting media differ from each other in the concentration and ratio of two auxin hormones: Indolo-3-acetic acid (IAA) and Indole-3-butyric acid (IBA). In vitro rooted plants obtained from the rooting phase were transferred to ex vitro conditions in a microbox with agri-perlite and a solution containing Murashige and Skoog (MS) basal medium at half concentration. With a culture medium containing the highest TDZ doses (H6) and combination with cytokinin (H8 and H9), the highest shoot percentage was obtained. After 3 months of in vitro culture, the highest shoot percentage was observed in the culture medium with 2 mL L^−1^ of BAP. The highest rooting percentage, roots numbers and root length were found when the culture medium was supplemented with 1 mL L^−1^ of IAA. The usage of agri-perlite and MS at half concentration, without PGR, allowed us to obtain a 99.1% survival rate. This micropropagation protocol is useful for obtaining virus-free plants and for the development of the brewery industry.

## 1. Introduction

The hop plant, *Humulus lupulus* L., is taxonomically categorized among the *Cannabaceae*; it is an herbaceous perennial dioecious species, although instances of monoicous exemplars have been found in some wild populations in North America [1]. Only female plants are used for cultivation, as they produce flowers, called cones. The cones contain bitter acids, flavonoids and essential oils, which are highly valuable for both the food and pharmaceutical industries [2,3,4].

Young shoots are consumed in many Mediterranean countries, either fresh or following a simple technological treatment [5], as they are rich in carotenoids, vitamins, organic acids and tocopherol, which confer antioxidant properties to the flowers [6,7,8,9,10,11,12].

Although wild hop plants are classified as lianous phanerophytes [13], cultivated hop plants behave as hemicryptophytes. Following harvesting of the cones, the shoots are cut to ground level and resprout the following spring [14].

The total world surface area cultivated by the hop plant is estimated to be around 100,151 hectares (2020), nearly 34% of which is on European soil [15], making Europe the center of the world hop market. In Italy, very little land is used for hop cultivation (40 ha) [15]; however, the emergence of artisanal breweries and increasing interest shown by producers for local hop plants would suggest a potential increase in the number of hectares dedicated to hop plants in the near future. A great deal of research is currently focusing on the development of competitive and sustainable brewing methods in the Mediterranean [16,17,18,19].

Despite increasing interest, cultivating hop plants in the Mediterranean is associated with certain challenges that must be overcome for more appreciable production. Factors related to photoperiod [20], climate conditions [21], crop care and the efficiency and occurrence of harvesting and post-harvest machinery [14] require specific attention. A further limitation to the extent of hop production in the Mediterranean is the provision of high-quality plant material that is suitable for propagation, which can deteriorate during transport [14,20,21,22,23,24]. Another critical issue is related to the introduction of non-certified plant material which could facilitate the introduction of noxious organisms on the plants, the packaging or cultivation substrates [23]. In response to increasing demand for hop plants in the Mediterranean area, development of the sector is linked to the creation of structures and methodologies suited to the production of plants with the required quality and safety features.

Traditionally, vegetative propagation is the most common method for multiplying hop plants. Parts of the root stock or herbaceous root cuttings without nodes, harvested from adult plants during the autumn or spring months, produce plantlets which are identical to the mother plant [23]. This propagated plant material can be planted directly into the soil, or initially into pots and subsequently transferred to the open field after 6–12 months [14]. Sexual reproduction, however, is used only in genetic improvement programs.

An alternative method to asexual reproduction is in vitro propagation. This can be useful when trying to increase the production of good-quality plants to satisfy the growing demand for plants from farmers. In vitro propagation is of interest for large-scale production of healthy and genetically uniform plants irrespective of the season [24].

This form of propagation is possible due to the ‘totipotency’ of the plant cells, which describes their ability to differentiate and acquire meristematic capacity [25,26]. With the exception of somaclonal variability, micropropagation is used not only to produce clonal plants, but also in the initial stages of other tissue culture processes in many plant species [27].

In hop plants, micropropagation can be used for the large-scale production of plantlets [28] and as a technique to eradicate viruses and ensure healthy plants [29]. Micropropagation in hop plants can be used for the safeguard of germplasm [30,31], genetic transformation [32] and ploidy manipulation [33].

Given the importance of the hop plant in the world, a number of protocols for in vitro propagation were developed over the years, proving the success of micropropagation in *H. lupulus* [23,33,34,35,36,37,38,39,40,41,42]. However, the development of a universal protocol which can be used for all species has not yet proved possible. Each species has a separate protocol, and there may also be different protocols for different cultivars within the same species. However, it would seem that all protocols agree on the fact that the in vitro propagation of hop plants requires the restriction of callus formation during the multiplication phase, as it is traditionally considered one of the factors which can lead to somaclonal variation [39,43].

The micropropagation technique adopted in this study involved multiplication using nodal segments with axillary buds, as this method is able to guarantee genetic stability in new plants [44].

In the literature, there is no research that simultaneously evaluates the effectiveness of the three different PGRs used in the multiplication phase. For this reason, the research in this paper assessed the response to in vitro multiplication and rooting, and the acclimatization of propagated hop plants (Cascade variety) to ex vitro conditions using different culture methods and variations in phytoregulator applications. In particular, different concentrations of plant growth regulator (PGR), different PGRs and their synergic effects were assessed. In vitro rooted plants were transferred to in vivo conditions without the use of PGR and using only agri-perlite.

## 2. Results

### 2.1. Effect of Culture Medium on Shoot Rates, Shoot Number, Shoot Length and Callus Percentage

The nine culture mediums (H1-H9) used during plant multiplication stage showed statistically significant differences (*p* ≤ 0.01) regarding all parameters in the study: shoot rate, number of shoots, shoot length and callus percentage (Table 1).

The explants placed in growing media H6, H8 (89%), H9 (86%) and H7 (83%) showed the highest shoot percentage, followed by those grown in H1 (81%) and H5 (79%). The lowest rates were obtained in culture medium H3 (53%).The media H1, H4 and H5 revealed the highest numbers of shoots with respect to the other media, with average values above three shoots. Also, for this parameter, the lowest value was obtained with medium H3.

The greatest shoot length was found on explants grown in media H8 and H9 (3.1 cm); however, these values did not differ statistically from shoot lengths obtained from plantlets grown in media H6 and H7 (3.0 cm). Lower lengths were recorded in plants grown in culture media H2 (2.3 cm), H5 (2.1 cm), H3 and H4 (2.0 cm) (Table 1).

The highest callus rates were recorded in treatment H1 (93%), not found to be statistically different from callus rates obtained in culture media H9 (91%), H7 and H8 (90%). Lower callus rates were obtained in plants grown in medium H3 (45%).

### 2.2. Effects of Culture Medium on Shoot Percentage, Shoot Number, Shoot Length, Rooting Rates, Root Number and Root Length

Considering the results obtained from the multiplication phase, the explants of treatment H6 were chosen to carry out subsequent in vitro rooting tests. The basal callus present in explants was removed to enhance rooting. The culture media (HR) used to evaluate rooting showed statistically significant differences regarding the following parameters: shoot length, rooting percentage, root number and root length. In contrast, statistically significant differences were not found for shoot percentage or shoot number (Table 2).

The longest shoots were found in treatments HR1 (2.51 cm) and HR4 (2.33 cm); however, no statistical differences were found in shoot lengths from culture media HR3 (2.07 cm) or HR5 (2.02 cm). The shortest shoot lengths were obtained from culture medium HR2 (1.30 cm).

**Figure 1:**
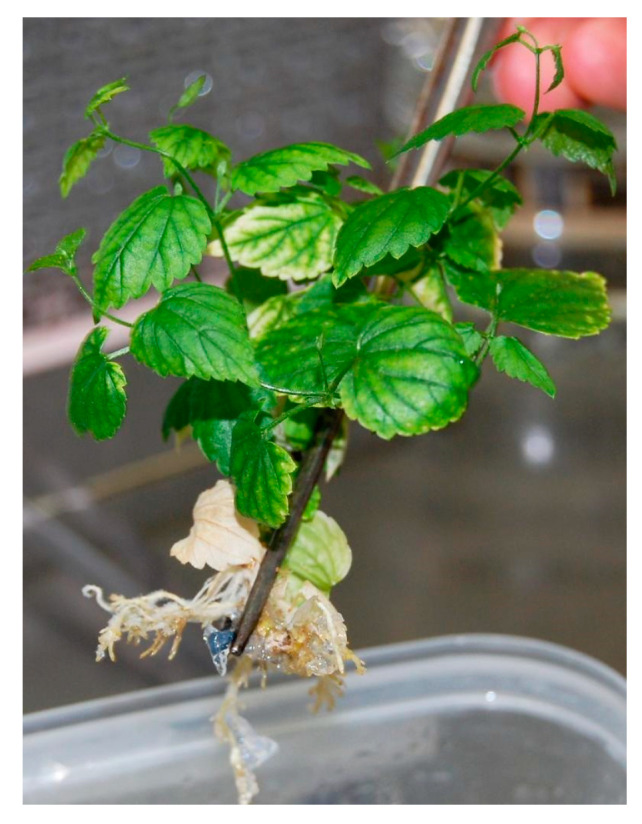
Plantlets obtained from in vitro rooting phase before next step.

The highest rooting rates (72%) were obtained, once again, in culture media HR1 and HR4. In contrast, the lowest percentages were recorded in culture media HR3 and HR5 (50%). The highest average root number was found in plants grown in media HR1, HR2 (7.2) and HR4 (6.1). A lower average number of roots were found in plants grown in media HR3 (4.2) and HR5 (3.1), with no statistical differences between the two. The highest average root lengths were obtained in culture medium HR1 (1.17 cm), while the lowest average length was found in culture medium HR5 (0.40 cm) (Figure 1).

### 2.3. Acclimatization

**Figure 2:**
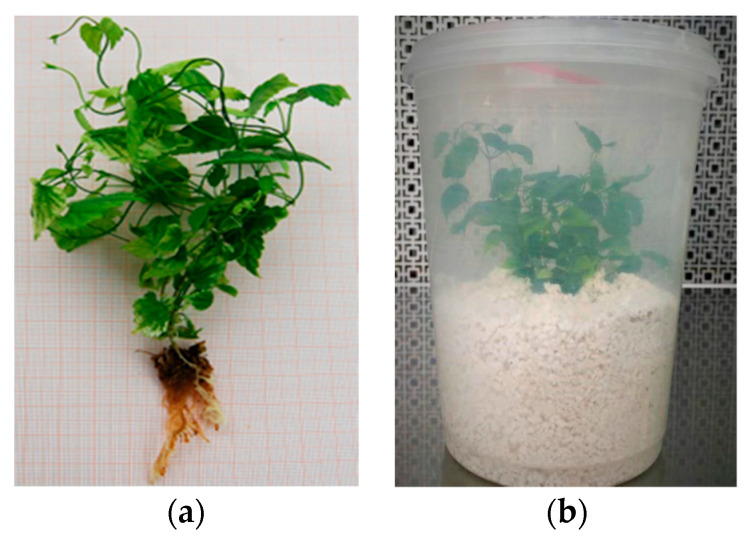
Acclimatization phase. (**a**) in vitro derived plants, before transplantation; (**b**) plants in sterile microbox with agri-perlite, during the acclimatization phase.

After thoroughly washing the root system with sterile distilled water to remove all solid residues of the culture medium, the plantlets obtained from the in vitro rooting process were incubated in sterile round microboxes (Figure 2a,b) containing sterile agri-perlite and a liquid solution at half-dose of MS salts and vitamins, under the same light, temperature and photoperiod conditions as the previous stages. After 30 days of growth, the survival rate was found to be 99.14%. The microboxes were then opened gradually and moved to a cold greenhouse so that the plantlets could be gradually acclimatized and transferred to the open field.

## 3. Discussion

Although found to be a fairly reliable method, as reported by Rossini et al. [14], the plant propagation of hops is restricted due to the limited availability of rhizomes.

In vitro tissue culture techniques have been tested for a wide range of species to ensure the reproduction of both genetically uniform, disease-free plants and endangered species [45]. As is the case for many crops, the main factors which limit industrial exploitation are the availability and quality of plant material. Rapid and efficient in vitro regeneration which minimizes somaclonal variation is fundamental in conventional breeding programs. It is potentially useful for providing clones with desirable traits free from pathogens. Specialized techniques were developed for many plants that allow the multiplication of isolated cells, tissues, and organs. These techniques are based on the ability of plant tissues and cells to undergo morphogenesis, resulting in whole organs or plants [46]. It is worth noting that many species or cultivars are recalcitrant to tissue culture or have slow or limited multiplication rates.

The composition of the culture medium is a key factor in tissue culture. Several studies on hops report plant regeneration from different types of explants, such as internodes, petioles and leaf portions [28,34,37,47,48,49,50]. Many authors have reported that internodal cuttings provide the most successful hop plant regeneration method [28,32,34,49,51,52,53]; however, it is also known that the regeneration capacity of the hop plant is highly dependent on genotype [28]. The primary aim of this study, therefore, was the induction of in vitro organogenesis from nodal segments of the hop shoots of the Cascade variety by testing the type and concentration of plant growth regulators in culture media and subsequent transfer to ex vitro conditions.

Regardless of the culture media, microcuttings grown in vitro retained their green color for the first 10 days, subsequently turning light brown and producing a yellow, spongy callus, which then covered the entire surface in contact with the culture medium. As reported by other authors [47,53], callus formation is the first sign of organogenesis. Twelve weeks after planting, ANOVA analysis revealed statistically significant differences between the culture media; in particular, the highest callus rates were found in the culture medium with the lowest dose of BAP, and in the culture media with various cytokinins in combination. It has been shown that exogenous occurrence of cytokinin and auxin in equal amounts can induce a callus [54]. The size of the intercellular connection generated in the callus (described by Thorpe [55]) probably determines the differentiation level that regulates the number of shoots per explant.

All culture media promoted shoot formation. Culture medium fortified with TDZ, both at the highest dose (H6) and in combination with the other cytokinins (H8 and H9), obtained the highest shoot rates. In agreement with Roy et al. [37] regarding the in vitro multiplication of hops, TDZ promotes better responses both when added alone and when in combination with other cytokinins.

Considering the medium fortified with TDZ alone (H6: 2 mg L^−1^), and comparing it with the abovementioned study, it is clear that shoot rates were much higher in our study (89% vs. 38%). TDZ is preferentially selected for in vitro propagation of many plant species as it causes high *BrCKX* and *BrIP* gene expression [56], leading to an outstanding ability to stimulate shoot proliferation [57,58,59]. Furthermore, as reported by many authors [60,61,62] regarding other species, TDZ can determine specific traits more efficiently when used in conjunction with other cytokinins or auxins. On this point, we would like to bring the reader’s attention to the high shoot rates recorded in our study in the culture media with TDZ in combination with other cytokines, such as BAP and mT (H8, H9 and H7).

Each nodal segment of a hop plant consists of a node with two buds which produce new shoots, leading to exponential multiplication [63]. In addition, explants from the nodal segment are less likely to contribute to somaclonal variation, as plantlet regeneration usually occurs without the intermediate callus stage, thereby preserving genetic fidelity [63]. After 3 months of in vitro growth, the medium with the highest number of shoots was supplemented with 2 mL L^−1^ of BAP (H4), followed by medium H1, supplemented with the lowest dose of BAP, and H5 supplemented with the highest dose of mT. As reported by Clapa and Harta [41] in a study on the use of four cytokinins for the in vitro multiplication of hops, 6-benzylaminopurine (BAP—the most commonly used plant growth regulator in propagation) was found to be the best, producing more shoots than the other cytokinins.

Meta-Topoline (mT) has been used successfully in place of 6-benzylaminopurine (BAP) in the micropropagation of some species [64,65]. IAA was added to the culture medium to induce rooting, not the growth of aerial parts. This same effect of IAA on shoot growth has also been reported in the in vitro culture of several species, such as pineapple [66] and peppermint [67], indicating that when added in low doses, it can promote shoot elongation.

Regarding shoot length, following three months of in vitro culture, the greatest length was obtained in the culture media combining TDZ + BAP (H8) and TDZ + mT (H9), followed by H6 with 2 mL L^−1^ of TDZ and H7 with 0.5 mL L^−1^ of BAP + 0.5 mL L^−1^ of mT. In agreement with Roy et al. [37], TDZ led to favorable results for average shoot length. As reported by Deepa et al. [68] and Guo et al. [69], for many species, a combination of TDZ with other phytohormones might be more effective than single use in both inducing more shoots and a higher shoot length. However, based on various studies reported in the literature, it has been shown that optimal levels of TDZ seem to vary according to plant species, propagation material and time spent in contact [37,68,69].

Callus formation can lead to somaclonal variation, a trait which is undesirable in a system designed to produce clonal material [70]. Therefore, taking into consideration the results of the different parameters examined in the treatments under comparison, i.e., those relating to the percentage of callus formation, the explants (following removal of the basal part) of treatment H6 were chosen to carry out subsequent in vitro rooting tests.

After 2 months of in vitro culture in the rooting media, there were clear signs not only of root system development but also of shoot growth, despite the media not receiving cytokinin supplements. The longest shoots were recorded in medium HR1 (2.51 cm) and HR4 (2.33 cm), media which differed in auxin type and amount administered. As reported in other studies [63,71], shoot development was induced by the auxin alone. This process can be attributed to cell wall acidification caused by auxin, which induces loosening of the cell wall and increased water and potassium uptake, leading to cell expansion. Auxin acts as a signal for cell division, elongation and differentiation, and plays an important role in apical dominance. In addition, shoot segmentation and the consequent disruption of apical dominance in plants leads to lower levels of auxin (which flows from the apex to the base of the plant), thus freeing the axillary bud from inhibition during development [37,41,61].

The highest rooting rates, root numbers and relative lengths were recorded when the culture medium was supplemented with 1 mL L^−1^ of IAA (HR1), not differing statistically from HR4 for both rooting rates and root numbers. Regarding root numbers, HR2 did not differ from HR1 either.

As is well known, rooting can be enhanced by rinsing the plant tissue in an auxin solution. Many authors [23,28,37,41,63,72] have carried out various types of auxin testing in in vitro rooting media of hop plants, showing rooting rates, root numbers and root lengths in line with our results. These studies, unlike ours, did not include the addition of auxin alone, only in combination with other PGRs. The results of our study could be considered more cost-effective in the rooting process.

The acclimatization of plantlets is a crucial step for the success of in vitro propagation. Many researchers are attempting to reduce the number of steps in the micropropagation process by integrating the rooting stage with the acclimatization stage in order to lower costs [73,74]. Due to a weak root system and the incomplete autotrophy of plants obtained through in vitro culture, acclimatization rates today are not high, relatively speaking, in particular when the substrates are composed of only organic material [75,76]. Plantlets (consisting of epigeal and hypogeal parts) from the culture medium HR1 were used for the evaluation of the subsequent acclimation stage.

**Figure 3:**
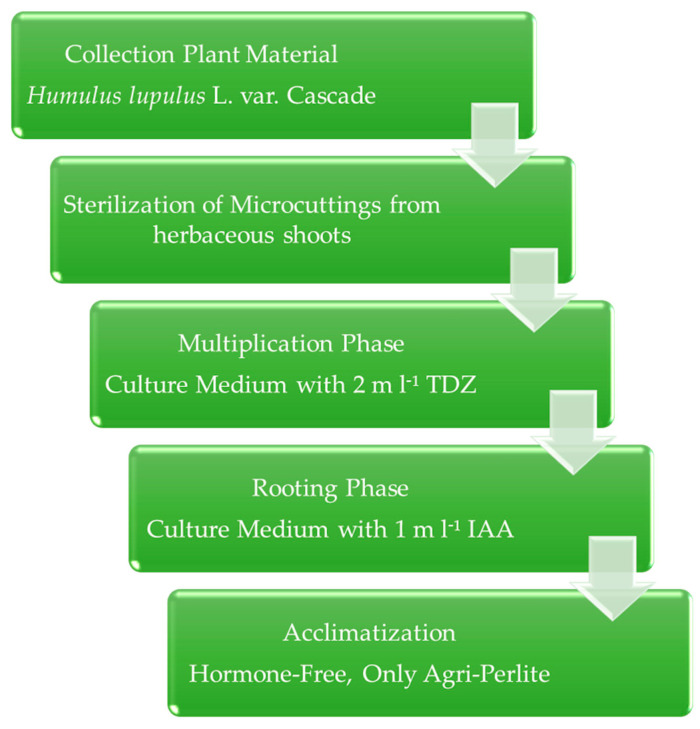
Scheme of the proposed protocol for *Humulus lupulus* L. var. Cascade propagation. TDZ: Thidiazuron; IAA: Indolo-3-acetic acid.

The use of agri-perlite with a half dose of MS salts and vitamins (without the addition of PGR), was found to be a good technique for the acclimatization of hop plants (Cascade variety) to ex vitro conditions. Our results agree with the study conducted by Clapa and Harta [41], achieving close to 100% acclimatization rates in the absence of PGR. The plantlets showed well-developed root systems which allowed better establishment and anchoring of the plants when transferred to the field. The protocol that allowed to obtain the best results is reported in Figure 3. From the 150 initial explants, 119 plantlets (79%) were achieved after the acclimatization phase.

## 4. Materials and Methods

### 4.1. Plant Material and Sterilization

Plant material of *Humulus lupulus* L. used in the trial was gathered during spring 2019 from the Cascade variety grown at a private farm in Aragona (AG), Sicily, Italy. The Cascade variety was developed in the United States by the U.S. Department of Agriculture (USDA) in 1956 and has been used on a commercial scale to produce plantlets for cultivation since 1976. This hop variety has high vigor, excellent yield, and distinctive aromatic characteristics with spicy notes of citrus, most notably grapefruit [77].

Microcuttings from herbaceous shoots of the plant were sterilized prior to in vitro culture. Sterilization involved the following steps: 45 min rinsing under running water to remove dust and phenolic substances; agitated in a 10% solution of water and detergent for 10 min; rinsing under running water for a further 5 min; finally, agitation in a 2% fungicide solution for 10 min. The sterilization procedure then continued under a laminar flow hood. The explants were immersed first in a 70% alcohol solution for 5 min and then in a 30% sodium hypochlorite solution (with the addition of a few drops of Tween 20) for 20 min. After removing the sodium hypochlorite solution, the explants were rinsed individually three times for 3 min with sterile distilled water.

### 4.2. In Vitro Propagation

**Figure 4:**
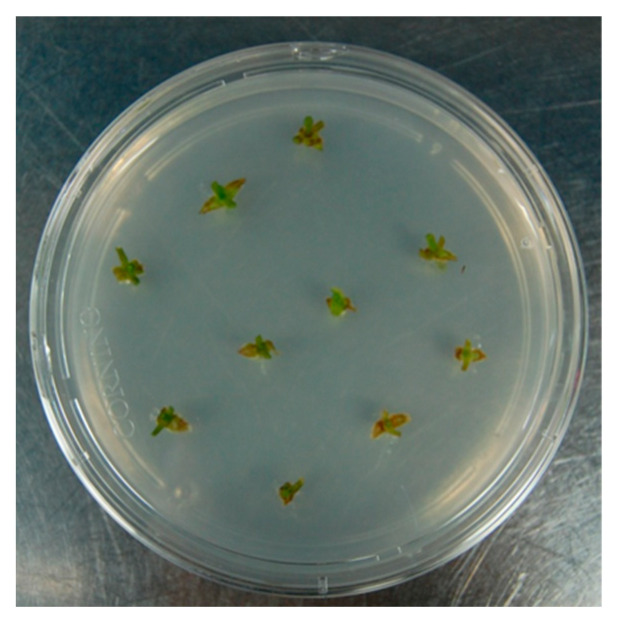
Single-node explants grown in vitro.

In order to identify the best PGR for multiplication, single-node explants were grown in vitro on nine different growth media (Table 3 and Figure 4).

The nine growth-media included macro- and micro-nutrients and vitamins from Murashige and Skoog (MS) [78] to which 6.3 mL L^−1^ of sucrose and 6.5 mL L^−1^ of maltose were added as a carbon source, 1 mL L^−1^ of IAA was added as auxin to all media and 14.5 mL L^−1^ of agar as a solidifying agent °C for 20 min. The culture media differed in the type, concentration and ratio of the cytokinin used. The cytokinin tested in this study were Benzylaminopurine (BAP), meta-Topoline (mT) and Thidiazuron (TDZ). In this study, a multiple comparison between hormones was used, without considering a control. All media were autoclaved at 0.11 MPa at 121 °C for 20 min. For each treatment, 15 Petri dishes were prepared with 150 single-node explants per treatment (10 explants per Petri dish).

### 4.3. In Vitro Rooting

Once the best growth medium for multiplication purposes was determined and a sufficient number of shoots were obtained, lateral binodal microcuttings were isolated for rooting tests. Five different culture media were tested by taking cuttings from the whole multiplication area (Table 4). 

The rooting medium and the multiplication medium did not differ in composition as regards micro and macronutrients, vitamins and carbon source, except for the PGR. However, the media differed in type, concentration and ratio of auxins tested. Auxins selected for the tests were indol-3-acetic acid (IAA) and indol-3-butyric acid (IBA). All media were autoclaved at 0.11 MPa at 121. Each treatment included 10 microbox trays, each containing 8 explants (a total of 80 explants per treatment).

### 4.4. Growth Conditions

For both the multiplication and rooting tests, Petri dishes and microbox trays were incubated at 27 ± 1 °C and placed under fluorescent lamps (Philips TLM 30w/84) with a photosynthetic photon flux intensity of 3 μmol m^−2^ s^−1^ and a 16-h photoperiod.

### 4.5. Acclimatization to Ex Vitro Conditions

Acclimatization was induced after 60 days of in vitro rooting. The plantlets (obtained from the best rooting medium) were removed from the growth microboxes and washed under running water to remove the culture medium completely. The plantlets were then placed in plastic containers (180 mL) filled with sterile agri-perlite, a half-dose of macro- and micro-nutrients, and MS vitamins. Plantlets were then transferred to a greenhouse where temperature and moisture were carefully monitored and controlled. To maintain moisture levels, each pot was covered with clear plastic film for 15 days. This was gradually removed over subsequent days. Once survival was established, the plantlets were then transferred to the open field.

### 4.6. Data Collection

Bacterial or fungal contamination was monitored from the first week of culture and throughout the entire crop period.

During the shoot stage, data on percentage of shoot (%); number of shoots per explant (no.); main-shoot length (cm); and percentage of callus formation (%: Percentage of shoots that had callus) were collected after 90 days.

After 60 days of in vitro culture, the following data were collected relating to the rooting stage: rooting (%); number of roots emitted (no.); main-root length (cm); percentage of shoot (%); number of shoots (no.); main-shoot length (cm); and callus formation (%; explants that generated a callus).

### 4.7. Statistical Analysis

All data were subjected to analysis of variance. The difference between the means was determined using Tukey’s test (*p* ≤ 0.05). Data analysis was performed using Minitab 19 software for Windows.

## 5. Conclusions

In this research, it was found that the different types of cytokinin and auxin, added in differing concentrations to the culture media, had a favorable effect on the Cascade variety of *H. lupulus*. The best results in terms of growth and shoot development were obtained by adding TDZ to the propagation medium and IAA to the rooting medium. Results of close to 100% acclimatization allowed good establishment of the crop in open-field conditions. The production of hop plant material using the micropropagation protocol developed in this study shows great economic potential to produce virus-free plants, encouraging the growth of a large-scale brewing industry.

## Figures and Tables

**Table 1:** Effect of culture medium used during the multiplication phase on shoot percentage, number of shoots, shoot length and callus percentage.

Culture Medium	Shoot Percentage [%]	Shoots [n.]	Shoot Length [cm]	Callus Percentage [%]
H1	81 b	3.1 ± 1.3 a	2.7 ± 1.0 b	93 a
H2	69 c	2.4 ± 0.8 bc	2.3 ± 0.6 c	79 b
H3	53 d	2.1 ± 0.6 c	2.0 ± 0.5 c	45 e
H4	71 c	3.2 ± 0.6 a	2.0 ± 0.4 c	59 d
H5	79 b	3.1 ± 1.2 a	2.1 ± 0.6 c	66 c
H6	89 a	2.5 ± 1.0 bc	3.0 ± 0.8 ab	70 c
H7	83 ab	2.6 ± 1.0 bc	3.0 ± 1.0 ab	90 a
H8	89 a	2.6 ± 1.0 bc	3.1 ± 0.9 a	90 a
H9	86 a	2.7 ± 0.9 b	3.1 ± 0.8 a	91 a
Significance	**	**	**	**

The composition of the multiplication culture medium is given in the Materials and Methods section. Values with different letters are significantly different at *p* ≤ 0.05. ** = significant at *p* ≤ 0.01.

**Table 2:** Effect of culture medium used during the rooting phase on shoot percentage, number of shoots, shoot length, rooting percentage, number of roots and root length.

Culture Medium	Shoot Percentage [%]	Shoots [n.]	Shoot Length [cm]	Rooting Percentage [%]	Roots [n.]	Root Length [cm]
HR1	90 a	2.1 ± 1.1 a	2.51 ± 1.8 a	72 a	7.2 ± 3.7 a	1.17 ± 0.6 a
HR2	94 a	2.2 ± 1.1 a	1.30 ± 1.2 b	58 b	7.2 ± 3.7 a	0.61 ± 0.3 c
HR3	82 a	2.1 ± 1.1 a	2.07 ± 1.7 ab	50 c	4.2 ± 3.4 b	0.56 ± 0.6 c
HR4	90 a	1.9 ± 1.3 a	2.33 ± 1.7 a	72 a	6.1 ± 3.6 a	0.78 ± 0.5 b
HR5	84 a	2.1 ± 1.3 a	2.02 ± 1.6 ab	50 c	3.1 ± 3.3 b	0.40 ± 0.3 d
Significance	n.s.	n.s.	**	*	**	**

The composition of the rooting culture medium is given in the Materials and Methods section. Values with different letters are significantly different at *p* ≤ 0.05; n.s. = not significant; * = significant at *p* ≤ 0.05; ** = significant at *p* ≤ 0.01.

**Table 3:** Composition of the culture medium used during the multiplication phase.

Components	H1	H2	H3	H4	H5	H6	H7	H8	H9
MS salts and vitamins ^1^	x	x	x	x	x	x	x	x	x
Sucrose (mL L^−1^)	6.3	6.3	6.3	6.3	6.3	6.3	6.3	6.3	6.3
Maltose (mL L^−1^)	6.5	6.5	6.5	6.5	6.5	6.5	6.5	6.5	6.5
IAA ^2^ (mL L^−1^)	1	1	1	1	1	1	1	1	1
BAP ^3^ (mL L^−1^)	1	-	-	2	-	-	0.5	0.5	-
mT ^4^ (ml L^−1^)	-	1	-	-	2	-	0.5	-	0.5
TDZ ^5^ (ml L^−1^)	-	-	1	-	-	2	-	0.5	0.5
Agar (g L^−1^)	14.5	14.5	14.5	14.5	14.5	14.5	14.5	14.5	14.5
pH					5.8				

^1^ Murashige and Skoog [78]; ^2^ IAA: Indolo-3-acetic acid; ^3^ BAP: 6-benzylaminopurine; ^4^ mT: meta-Topoline; ^5^ TDZ: Thidiazuron. x = presence of Murashige and Skoog (MS) basal medium with salts and vitamins.

**Table 4:** Composition of the culture medium used during the multiplication phase.

Components	HR1	HR2	HR3	HR4	HR5
MS salts and vitamins ^1^	x	x	x	x	x
Sucrose (mL L^−1^)	6.3	6.3	6.3	6.3	6.3
Maltose (mL L^−1^)	6.5	6.5	6.5	6.5	6.5
IAA ^2^ (mL L^−1^)	1	2	-	-	0.5
IBA ^3^ (mL L^−1^)	-	-	1	2	0.5
Agar (g L^−1^)	14.5	14.5	14.5	14.5	14.5
pH			5.8		

^1^ Murashige and Skoog [78]; ^2^ IAA: Indolo-3-acetic acid; ^3^ IBA: Indole-3-butyric acid. x = presence of Murashige and Skoog (MS) basal medium with salts and vitamins.

## Data Availability

Data sharing not applicable.
